# Metabolites and Biological Activities of *Thymus zygis*, *Thymus pulegioides*, and *Thymus fragrantissimus* Grown under Organic Cultivation

**DOI:** 10.3390/molecules23071514

**Published:** 2018-06-22

**Authors:** Andrea F. Afonso, Olívia R. Pereira, Mónica Válega, Artur M. S. Silva, Susana M. Cardoso

**Affiliations:** 1 Department of Chemistry & QOPNA, University of Aveiro, 3810-193 Aveiro, Portugal; andrea@ipb.pt (A.F.A.); mvalega@ua.pt (M.V.); artur.silva@ua.pt (A.M.S.S.); 2 Public Health Laboratory of Bragança, Local Health Unit, Rua Eng. Adelino Amaro da Costa, 5300-146 Bragança, Portugal; 3 Department of Diagnostic and Therapeutic Technologies, Polytechnic Institute of Bragança, School of Health Sciences, Av. D. Afonso V, 5300-121 Bragança, Portugal; oliviapereira@ipb.pt; 4 Centro de Investigação de Montanha (CIMO), Instituto Politécnico de Bragança, Campus de Santa Apolónia, 5300-253 Bragança, Portugal

**Keywords:** *Thymus*, thyme, phenolic compounds, nutrients, antioxidant, antibacterial activity

## Abstract

*Thymus* plants are marketed for diverse usages because of their pleasant odor, as well as high nutritional value and wealth of health-promoting phytochemicals. In this study, *Thymus* *zygis*, *Thymus* *pulegioides*, and *Thymus* *fragrantissimus* grown under organic cultivation regime were characterized regarding nutrients and phenolic compounds. In addition, the antioxidant and antibacterial properties of these species were screened. The plants were particularly notable for their high K/Na ratio, polyunsaturated fatty acids content and low omega-6/omega-3 fatty acids ratios, which are valuable features of a healthy diet. Caffeic acid and/or its derivatives, mainly rosmarinic acid and caffeoyl rosmarinic acid, represented the majority of the phenolic constituents of these plants, although they were less representative in *T. pulegioides*, which in turn was the richest in flavones. The latter species also exhibited the highest antioxidant capacity (DPPH^●^ EC_50_ of 9.50 ± 1.98 μg/mL and reducing power EC_50_ of 30.73 ± 1.48 μg/mL), while *T. zygis* was the most active towards Gram-positive and Gram-negative bacteria. Overall, the results suggest that the three thyme plants grown in organic farming are endowed with valuable metabolites that give them high commercial value for applications in different industries.

## 1. Introduction

*Thymus* plants, belonging to the Lamiaceae family, represent a large botanical genus well known in the West Mediterranean region. Due to their high capacity to adapt to extreme climate conditions concerning temperature and water supply, the plants can often be found in rocks or stones growing in cold and arid conditions [1]. In general, they appear as perennial and subshrubs or shrubs 10 to 30 cm tall, with small and simple leaves, a quadrangular stem erect to prostate, ramified and prostrated branches, and big clusters of small pink, white, cream or purple flowers [2,3].

Because of its easy growth and the wide variety of *Thymus*-derived products that can be used by diverse industries, the cultivation of thyme species for commercial purposes has increased greatly in recent decades [4]. Special interest is given to organic farming, since this is recognized as a sustainable agricultural system. Indeed, organic agriculture has developed rapidly worldwide in recent years, being presently practiced in approximately 120 countries. At the level of the European Union, the area under organic production increased by about 70% in the last decade, reaching about 11 million hectares [5]. Moreover, medicinal and aromatic plants represent one of the top 10 crops cultivated under the organic regime. Organically grown medicinal and aromatic products are readily accepted in global markets and command higher prices than those grown with chemical inputs [6]. Indeed, crops grown under an organic regime are considered healthier by consumers, since this agriculture practice restricts the use of synthetic fertilizers, pesticides, and herbicides [7,8]. Moreover, there is evidence that organic agriculture can lead to more valuable products. In this context, the concentrations of a range of polyphenolics such as phenolic acids, flavanones, stilbenes, flavones, flavonols, and anthocyanins were found to be substantially higher in organic crops/crop-based foods in several studies [9]. In particular, Sousa et al. [10] showed that the levels of phenolic compounds in leaves of tronchuda cabbage from organic culture had higher amounts of phenolics, probably due to the interference of mineral fertilizers and pesticides with the biosynthetic pathway of phenolic compounds.

Cultivated *Thymus* plants can be commercialized for a wide range of applications. In fact, *Thymus* are amongst the main aromatic plants used as culinary ingredients for fish, meat, salad and vegetable dishes [11,12]. Also, dried thyme leaves are used for teas as well as in other several products such as lotion, bath soap, and toothpaste [1,13]. In addition, thyme-derived products are applied in traditional medicine to treat respiratory and throat ailments, and skin problems such as oily skin, sciatica, acne, dermatitis, parasite affections, eczema, fungal infections, and insect bites, among others [14]. Moreover, thyme essential oil is a globally respected commodity and is currently used in many industries such as food, pharmaceuticals, personal health care, detergents, and insecticides [1,15].

The widespread application of *Thymus* plants has long been associated with their pleasant taste/odor, but at present, highly valuable products of these plants are closely associated with their high nutritional value and/or predominance of bioactive compounds. Among the latter, essential oils have undoubtedly been the most exploited health promoters of *Thymus*, being recognized for their antimicrobial, preservative, antispasmodic, diuretic, antihypertensive, and calming properties [12,16,17]. Although less studied, a growing number of investigations have been focused on the potential biological activities of extracts rich in phenolic compounds, and their prospective for application as high-value products [16,17].

The present study aims to evaluate the nutritional composition of three economically important *Thymus* species (*Thymus zygis*, *Thymus pulegioides*, and *Thymus fragrantissimus*) (Figure S1) cultivated in an organic farming regime, as well as to establish the phenolic profile and biological potencies (antioxidant and antimicrobial) of their respective decoctions. As far as we know, this is the first work focusing on organically grown *T. zygis*, *T. pulegioides*, and *T. fragrantissimus* plants. One must also remark that the chemical composition and/or biological potentials of these botanical species remain poorly studied, even for wild plants. In this regard, Fernandes et al. [18] previously reported the nutritional value of a wild *T. pulegioides* collected in the northeast of Portugal. In addition, these authors and Kindl et al. [19], also evaluated the antioxidant potential of methanolic or hydroethanolic extracts of wild *T. pulegioides* (in northeast Portugal and Croatia) against oxidative events.

## 2. Results and Discussion

### 2.1. Nutritional Composition

The nutritional profile of the three *Thymus* species cultivated under an organic farming regime is shown in Table 1. The dried plants were essentially rich in carbohydrates, varying from 78 to 87% dw, while protein, fat, and ash contents were in the ranges of 3.6–10.5%, 2.3–4.5%, and 5.0–8.2%, respectively. The levels of these macronutrients in *T. pulegioides* were 81.2, 8.3, 2.3, and 8.2 g/100 g dw, respectively, which, compared to those reported by Fernandes et al. [18] for a wild *T. pulegioides* collected in the northeast of Portugal (89.4, 5.5, 0.2, and 4.9 g/100 g dw, respectively), presented higher non-carbohydrate macronutrients. To our knowledge, there are no previous data on the nutritional value of *T. zygis* and *T. fragrantissimus* plants.

*T. pulegioides* was also the richest species with respect to its mineral content. However, it is worth mentioning that all three plants had high levels of Ca (0.57–1.04 g/100 g dw) and K (1.23–2.19 g/100 g dw), but modest Na amounts, thus representing high K/Na suppliers, which is of great importance when aiming to compensate for the modern Western diet, which is typically rich in NaCl. This ratio was particularly high in *T. pulegioides*, which, due to its richness in K, was established at 294, a fact that can give the plant enormous potential for application in functional foods directed to cardiovascular health claims.

**Figure 1:**
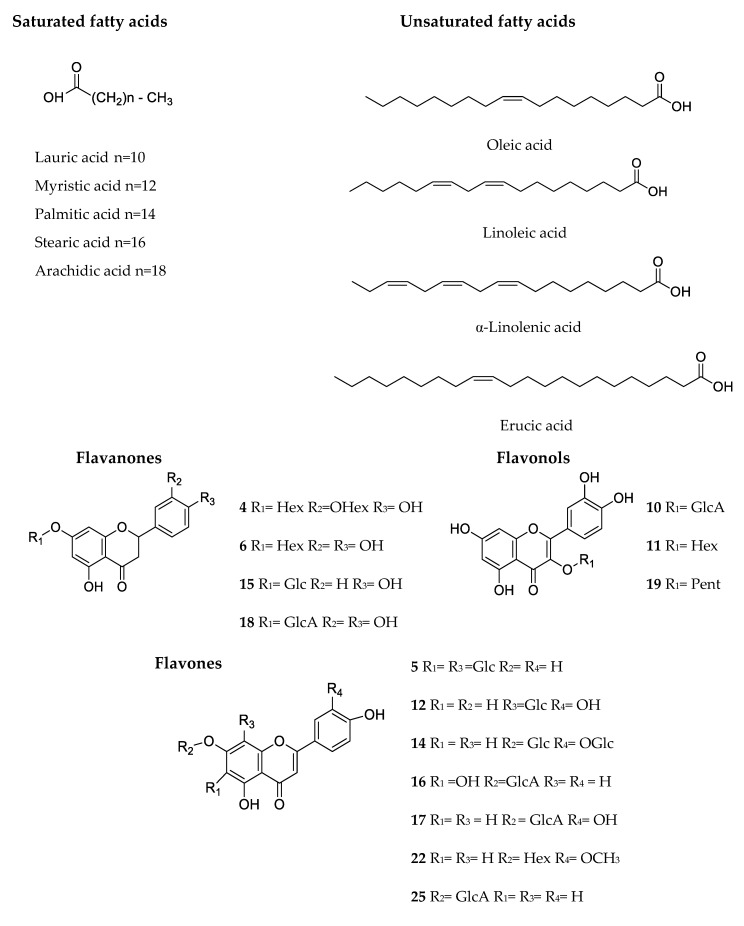

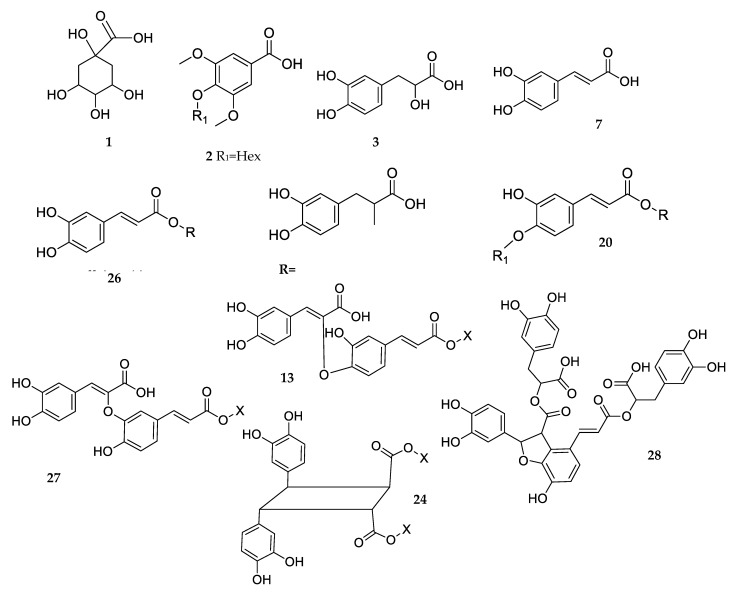
Chemical structures of fatty acids and phenolic compounds identified in *Thymus zygis*, *Thymus pulegioides*, and *Thymus fragrantissimus*. Numbers in the figure correspond to the UHPLC-DAD-ESI-MS^n^ peaks of Figure 2.

Regarding fatty acids, the results indicated that thyme plants were richer in unsaturated fatty acids (UFA), which represented 69–74%, whereas saturated fatty acids (SFA) represented 14 to 25% of the total fatty acids. Among UFA, polyunsaturated fatty acids (PUFA) were the main representatives, ranging from 48 to 49% in *T. zygis* and *T. pulegioides*, to 55% in *T. fragrantissimus*. Overall, PUFA comprised linoleic acid (C18: 2n6c) and α-linolenic acid (C18: 3n3), while monounsaturated fatty acids (MUFA) fraction was composed of oleic acid (C18: 1n9c) and erucic acid (C22: 1n9) (structures in Figure 1).

Although we found several SFA, palmitic acid (C16: 0) was by far the most abundant in the three samples, accounting for 18–24% of the fatty acids. Because of that, the PUFA/SFA ratio of the thyme plants was considerably high (1.62–1.83). This is particularly relevant when one examines the recommendation of the World Health Organization for the consumption of PUFA-rich foods as part of a healthy lifestyle, aiming at cardiovascular protection [20,21]. Moreover, it must be noted that the omega-6/omega-3 fatty acids ratios fall within the recommended values (<10) [22], and those of the *T. fragrantissimus* and *T. pulegioides* species (0.54 and 0.75, respectively) assume relevance due to its low value, which is believed to be associated with the prevention of many chronic diseases, including inflammatory bowel disease, rheumatoid arthritis, asthma, kidney disease, and several other inflammatory conditions [21,23].

Regardless of this generic similarity, specific features could be found among the plants. Linoleic acid was the most abundant fatty acid in *T. zygis*, while α-linolenic acid prevailed in the other species, with percentages of 28% and 36% in *T. pulegioides* and in *T. fragrantissimus*, respectively. Fernandes et al. [18] have found similar results for *T. pulegioides* (37% of α-linolenic acid) in a wild *T. pulegioides* plant. Nevertheless, the present data point to a lower prevalence of SFA and higher amounts of MUFA compared to that study. Naturally, differences may be related to multiple factors, including growth conditions (organic farming versus wild).

### 2.2. Phenolic Characterization of Thymus Decoctions

The yield range of the decoctions extracts of the three *Thymus* species was between 12% and 25%, with minimum and maximum values corresponding to *T. zygis* and *T. pulegioides*, respectively (Table 2).

The decoction of *T. pulegioides* also showed a superior content of total phenolic compounds when compared to the others (391 versus 287–288 μg GAE/ mg extract, *p*-values < 0.01) or to decoctions of *Thymus herba*-*barona*, *Thymus pseudolanuginosus*, and *Thymus caespititius* (236–293 μg GAE/mg of extract) [24], or even to aqueous extracts obtained from *Thymus serpyllum* at 50 and 100 °C (79 and 91 μg GAE/mg extract, respectively) [25]. Moreover, the extraction yield of this decoction was close to the one reported by other authors for methanol or 70% ethanol extracts of *T. pulegioides* wild plants (24.6 and 22.5, respectively) and its phenolic richness was superior to the latter (391 versus 210 μg GAE/mg extract) [18,19].

**Figure 2:**
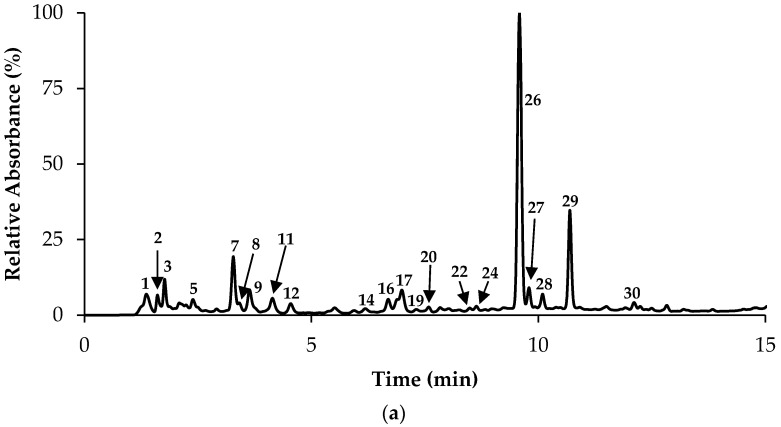

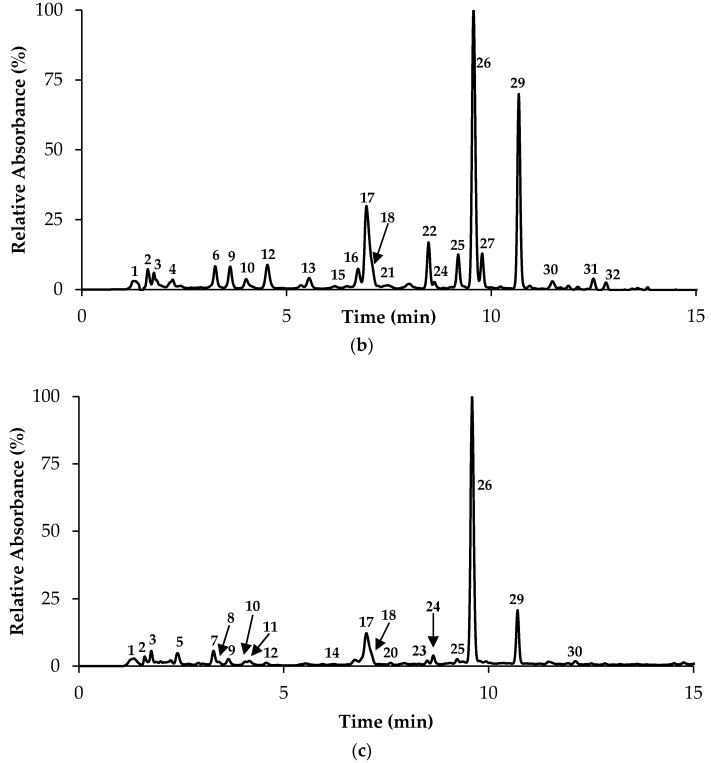
Chromatographic representation of *T. zygis* (**a**), *T. pulegioides* (**b**), and *T. fragrantissimus* (**c**) aqueous extracts at 280 nm. Numbers in the figure correspond to the UHPLC-DAD-ESI-MS^n^ peaks described in Table 3.

The phenolic profiles of *T. zygis*, *T. pulegioides* and *T. fragrantissimus* decoctions were evaluated using UHPLC-ESI-DAD-MS^2^/MS (Figure 2, Table 3). Moreover, the extracted ion chromatogram (EIC) and MS/MS spectrum of the main identified compounds are shown in Figure S2. Consistent with previous studies on *Thymus* polar extracts [3,24], these were mainly rich in rosmarinic acid (peak 26, UVmax at 290 and 328 nm, and [M − H]^−^ at *m*/*z* 539). Interestingly, its levels (62–82 µg/mg extract, Table 3) were higher than those previously found for decoctions of *T. herba*-*barona*, *T. pseudolanuginosus*, and *T. caespititius* (40–56 µg/mg extract) [24], as well as for hydroethanolic extracts of *Thymus x citriodorus* (10 µg/mg extract) [26] and methanolic extracts of *Thymus praecox* (15 mg/g dry weight) [27]. The same tendency was found for caffeoyl rosmarinic acid isomers (MW 538, eluted in peaks 27, 29, 30, 31 and 32), which amounted from 16 to 63 µg/mg extract in these species, whereas they were only vestigial in *T. pseudolanuginosus* and *T. caespititius* or up to 15 µg/mg extract, in *T. herba*-*barona* decoctions [24]. Apart from that, levels of caffeoyl rosmarinic acid in hydroethanolic extracts of *T. x citriodorus* were reported to only reach about 2 µg/mg extract [26]. Notably, caffeic acid and its derivatives (rosmarinic acid, caffeoyl rosmarinic acid, salvianolic acid I/H) represented most of the phenolic components in *Thymus* aqueous extracts (*p*-values < 0.01), although they were less representative in *T. pulegioides* (64% of total phenolic compounds) when compared to the remaining species (close to 80%).

In turn, the *T. pulegioides* decoction was the richest in flavones (55.62 ± 1.05 µg/mg extract, *p*-values < 0.001), represented mainly by luteolin-*O*-glucuronide (peaks 17 and 21, [M − H]^−^ at *m*/*z* 461→285), luteolin-*C*-glucoside (peak 12, MW 448), chrysoeriol-*O*-hexoside (peak 22, [M − H]^−^ at *m*/*z* 461→299, 285) and apigenin-*O*-glucuronide (peak 25, MW 446, UVmax at 267 and 334). Overall, levels of flavones in *T. pulegioides* decoction were higher than those previously found in *T. herba*-*barona* and *T. caespititius* (22 and 32 µg/mg extract), but lower than *T. pseudolanuginosus* (73 µg/mg extract) [24].

The extract of this species was also noticeable due to its greater amounts of flavanones (23.68 ± 0.57 µg/mg extract, with statistically significant differences, *p*-values < 0.001), mainly comprised by eriodictyol-*O*-hexoside (factions 6 and 9, UVmax 283 nm, [M − H]^−^ at *m*/*z* 449→287), naringenin-*O*-glucoside (peak 15, UVmax 283 nm, MW 434 and [M − H]^−^ at *m*/*z* 433→271), that overall accounted for 19.93 ± 0.46 µg/mg extract. Please note that the compounds described herein (caffeic acid derivatives and flavonoids, including glycosidic forms) were previously detected in aqueous or methanolic/ethanolic extracts of other *Thymus* species [3,26].

### 2.3. Antioxidant Activity

It is widely known that the antioxidant capacity of plant extracts are closely associated with phenolic components, which might interact with free radicals through electron or hydrogen donation [28]. In this study, the antioxidant potency of *Thymus* decoctions was screened by two generalized assays, namely the DPPH^●^ scavenging method and reducing power, which respectively evaluate the ability to trap the synthetic free radicals DPPH^●^ and to reduce ferric ion (Fe^3+^) to ferrous ion (Fe^2+^). In general, the extracts presented a high antioxidant capacity, since EC_50_ values were up to twice as high as the commercial reference compounds (Table 4). Among the three extracts, *T. pulegioides* had the lowest DPPH^●^ EC_50_ value (9.50 ± 1.98 μg/mL), which corresponded to about 1.4-fold of ascorbic acid (EC_50_ = 6.9 ± 0.5), while *T. fragantissimus* and *T. zygis* were established at 13 μg/mL. This fact shows the greater ability of *T. pulegioides* to scavenge the free radical DPPH^●^ in comparison to the other two plant species and can possibly be associated with its higher phenolic contents. Interestingly, regardless of the higher ferric ion reduction tendency (EC_50_ = 30.73 ± 1.48 μg/mL), the superiority of this extract was not so clear, suggesting that non-phenolic components might also play a relevant role in this reaction.

The high antioxidant ability herein described for *T. pulegioides* decoctions exceeded that previously described for the methanolic extract from a wild *T. pulegioides* plant (EC_50_ = 680 ± 30 and 490 ± 30 μg/mL DPPH^●^ and reducing power assays, respectively) [18]. More recently, Kindl et al. [19] found promising results for an hydroethanolic extract of wild Croatian *T. pulegioides* plants, reporting DPPH^●^ EC_50_ = 4.18 ± 0.02 μg/mL and reducing power EC_50_ = 11.39 ± 0.07 μg/mL, which corresponded to about 1.7–2.5 times those of the reference commercial compounds [19]. To our knowledge, there are no literature data regarding the antioxidant abilities of aqueous extracts from *T. zygis*, *T. pulegioides* or *T. fragantissimus* origin. Still, the high abilities reported herein for decoctions are in line with those previously described by us for *T. herba*-*barona*, *T. pseudolanuginosus*, and *T. caespititius* aqueous extracts [24]. However, Baharfar et al. [29] also observed that a *Thymus kotschyanus* aqueous extract could request DPPH^●^ with half the potency of ascorbic acid.

### 2.4. Antibacterial Activity

In opposition to *Thymus* essential oils, the antibacterial effects of polar extracts from *Thymus* plants have been scarcely exploited. In the present study, we evaluated the inhibitory activities of *T. zygis*, *T. pulegioides* or *T. fragantissimus* aqueous extracts against the Gram-positive bacteria *Staphylococcus aureus* and *Staphylococcus epidermidis*, and the Gram-negative bacteria *Salmonella typhimurium*, *Escherichia coli*, and *Pseudomonas aeruginosa*, through the broth microdilution assay.

Interestingly, *T. zygis* decoctions were the most active among the three plant samples. As the levels of phenolic compounds in this decoction were below those of *T. pulegioides*, the results suggest that phenolic components do not lead this bioactivity. *Staphylococcus aureus* was the most sensitive species to thyme decoctions, with its growth and viability being inhibited at 1.13, 3.75, and 5.75 mg/mL for *T. zygis*, *T. fragantissimus*, and *T. pulegioides*, respectively (Table 4). The results, combined with those previously reported for *T. herba-barona, T. pseudolanuginosus*, and *T. caespititius* allow us to conclude that, among the six aqueous extracts of thyme [24], *T. herba*-*barona* and *T. zygis* (MIC/MBC of 0.6 mg/mL and of 1.13, respectively) are the two most active towards *Staphylococcus aureus*. Overall, MIC and MBC for the six thyme species against this bacterium were established in the range of 0.6 to 5.75 mg/mL, which is in accordance with the reported data for thyme extracts of distinct origins. In this context, Benbelaïd et al. [30] reported an MIC value of 1.0 mg/mL for an aqueous extract of *Thymus lanceolatus*, while MIC values of 6.25 mg/mL, 5.0 mg/mL, and 0.5 mg/mL were previously reported for ethanolic extracts of *Thymus vulgaris*, and methanolic and ethanolic extracts of *Thymus capitatus* [31,32,33].

Among the tested Gram-negative bacteria, *S. typhimurium* was the most susceptible to *Thymus* extracts. Both the growth and viability of *S. typhimurium* were inhibited at 4.5 mg/mL by *T. zygis* and at 7.5 mg/mL by *T. fragrantissimus*. Instead, *T. pulegioides* inhibited bacterial growth at 5.75 mg/mL, but its viability was only affected at higher levels (MBC 11.5 mg/mL). Combined with our previous results for *T. herba-barona*, *T. pseudolanuginosus*, and *T. caespetitius* decoctions (MIC in the range of 3.5–7 mg/mL) [24], it may be suggested that, to inhibit *S. typhimurium*, aqueous extracts of thyme should be used in high concentrations. These results are also consistent with those from other authors. In particular, Benbelaïd et al. [30] reported a MIC value of 4.0 mg/mL when *S. typhimurium* was treated with a water extract from *T. lanceolatus*. Values of the same magnitude were reported for a methanolic extract of *T. capitatus* (MIC = 6 mg/mL) [32], or hydroethanolic extracts from *T. vulgaris* and *Thymus caramanicus* (MIC values of 12.5 and 2.6 mg/mL, respectively) [31].

In turn, *P. aeruginosa* and *E. coli* were even more resistant to thyme extracts, albeit at different levels. While *P. aeruginosa* growth was inhibited by all samples (MIC values of 4.5, 5.75, and 7.5 mg/mL for *T. zygis*, *T. pulegioides*, and *T. fragantissimus*, respectively), only the latter species negatively impacted *E. coli* growth at 7.5 mg/mL. Moreover, *T. pulegioides* was able to kill *P. aeruginosa* (MBC = 11.5 mg/mL), while no effect on the viability of *E. coli* was observed. *Thymus* ethanolic extracts have been previously demonstrated to inhibit *P. aeruginosa*, with MIC values of 2.0 mg/mL (*T. capitatus*) [33] and 0.25 mg/mL (*T. lanceolatus*) [30], but no data on their ability to kill this bacterium were delivered. Likewise, ethanolic extracts of *T. capitatus*, *T. vulgaris*, *T. caramanicus*, and *T. lanceolatus* were shown to inhibit the growth of *E. coli* strains (MIC in the range of 0.5–8 mg/mL) [31,33] although no information was given about their effects on the viability of this bacterium.

## 3. Materials and Methods

### 3.1. Chemicals

Rosmarinic acid, apigenin-7-*O*-glucoside, luteolin-7-*O*-glucoside and eriodictyol-7-*O*-glucoside were obtained from Extrasynthese (Genay, France). Gallic acid, nisin, ascorbic acid and 2,6-Di-*tert*-butyl-4-methylphenol and the fatty acids methyl ester (FAME) reference standard mixture 37 (fatty acids C4 to C24; standard 47885-U) from Supelco (Bellefonte, PA, USA) were obtained from Sigma Chemical Co (St. Louis, MO, USA). Folin–Ciocalteu reagent, Na_2_CO_3_, formic acid and ethanol were purchased from Panreac (Barcelona, Spain). *n*-hexane, methanol, and acetonitrile with HPLC purity were purchased from Lab-Scan (Lisbon, Portugal). Mueller–Hinton agar was obtained from VWR, Prolabo Chemicals (Radnor, PA, USA). Water was treated in a Direct-Q ^®^ water purification system (Merck Life Science, Darmstadt, Germany).

### 3.2. Plant Cultivation and Material

The *T. zygis*, *T. fragantissimus*, and *T. pulegioides* species have been cultivated under an organic regime by Ervital (Mezio-Castro Daire, Portugal, GPS coordinates 40.976351, −7.903492). The cultivation soil was of granitic origin, medium to coarse in texture. It was moderately acidic (pH in water 5.6), with an organic matter content of 3.9%, with low levels of extractable phosphorus (18 ppm) and high levels of extractable potassium (110 ppm). During the time of cultivation (April 2014–June 2015), local temperatures ranged from 2 °C to 27 °C. Minimum values were registered in winter (November 2014 to February 2015, 2 °C–12 °C), while temperatures in the spring varied between 11 °C to 22 °C and reached a maximum of 27 °C in June 2015. Relative humidity varied from 2% to 90% with 29% on average, while the average total precipitation was 75 mm (65–94 mm).

No chemical or bacteriological treatment was applied to the plants. Their appearance and behavior were regularly monitored to ensure their health and the absence of pathogens. Whenever appropriate, weeds were removed from the surrounding soil.

The aerial parts of each individual *Thymus* (flowers, leaves, and stems) were collected and dried in a ventilated incubator at 20 to 35 °C for three to five days. After drying, the plants were transported in Kraft paper-type bags to the lab, where they were kept frozen until use.

### 3.3. Extraction of Phenolic Compounds

The extraction of phenolic compounds was performed according to the method described by Ferreira et al. [34], with adaptations. Briefly, 0.5 mm mesh powder of the aerial parts (flowers, leaves, and stems) of *T. zygis*, *T. fragantissimus*, and *T. pulegioides* (5 g) were extracted for 15 min using a decoction 20:1 (5 g in 100 mL of water), filtrated, and concentrated using a rotary evaporator. The concentrate was defatted with *n*-hexane (1:1 *v*/*v*) and freeze-dried. This procedure was performed in three independent assays.

### 3.4. Nutritional Value

The chemical composition of samples was determined according to AOAC methods [35]. Crude protein content (N × 6.25) of the samples was estimated through the macro-Kjeldahl method. Ash content was determined by incineration in a muffle furnace at 550 °C for 6 h and gravimetric quantification. Crude fat was obtained by Soxhlet extraction with light petroleum for 8 h, followed by filtration through a 0.2 µm nylon filter, solvent removal in a rotary evaporator, and drying, followed by gravimetric quantification. Total carbohydrates were calculated by difference. Calorific content was calculated according to the following equation: Energy (kcal) = 4 × (g protein + g carbohydrates) + 9 × (g fat) [18].

For mineral determination, approximately 1 g of ash was digested with nitric acid. After digestion, samples were filtered and the volume adjusted to 100 mL with ultrapure water. Minerals (Na, K, Ca, Mg, Fe, Mn, Cu, and Zn) were quantified in a Perkin Elmer Analyst 100 flame atomic absorption spectrometer (Villepinte, France) equipped with a single hollow cathode lamps for each element and an air-acetylene burner [36].

Fatty acids were analyzed by transesterification of the fat fraction in the presence of sodium methoxide. The crude fat (1 g) was placed in a 15-mL glass tube and rod crushed and dissolved in 6 mL of *n*-heptane. Next, 400 μL of 0.2 M sodium methoxide were added to each sample and the tube was vigorously stirred in a vortex for 1–2 min at room temperature, allowing the formation of fatty acid methyl esters (FAME) from triglycerides. After decanting, sample supernatants were filtered through a 0.2 μm nylon filter from Milipore and injected in the gas chromatography system. Fatty acids were analyzed in a DANI 1000 gas chromatographer (GC) equipped with a split/splitless injector and a flame ionization detector (FID), following the method used by Fernandes et al. [18]. The identification was performed by comparing the relative retention times from samples with FAME peaks (standard mixtures). The results were recorded and expressed as a relative percentage of each fatty acid.

### 3.5. Identification and Quantification of Phenolic Compounds

The total phenolic content of each *Thymus* extract was determined according to the adapted Folin–Ciocalteu colorimetric method, as described by Pereira et al. [37]. The individual phenolic compounds were identified through UHPLC-DAD-ESI-MS^n^ analysis of extracts (5 mg/mL), as described by Afonso et al. [24]. Elution was carried out with a mixture of 0.1% (*v*/*v*) of formic acid in water (solvent A) and acetonitrile (solvent B) and the flow rate used was 0.2 mL/min^–1^, following the same program. UV–Vis spectral data for all peaks accumulated in the range 200–600 nm and the chromatographic profiles were recorded at 280, 320, and 340 nm. The mass spectrometer used was a Thermo LTQ XL (Thermo Scientific, San Jose, CA, USA) ion trap MS, equipped with an ESI source with Thermo Xcalibur Qual Browser software. The instrument was operated in negative-ion mode and the full scan covered the mass range from *m*/*z* 100 to 2000. Nitrogen above 99% purity was used and the gas pressure was 520 kPa (75 psi). ESI needle voltage set at 5.00 kV and an ESI capillary temperature of 275 °C. CID–MS/MS and MS^n^ experiments were simultaneously acquired for precursor ions using helium as the collision gas with collision energy of 25–35 arbitrary units.

For quantitative experiments, the limits of detection (LOD) and quantification (LOQ) were calculated from the parameters of the calibration curves obtained by injection of known concentrations of the exact or structurally related standard compounds represented in Table S1.

### 3.6. Antioxidant Activity

#### 3.6.1. DPPH^●^Scavenging Test

The scavenging capacity of different concentrations (0.05–0.8 mg/mL) of *T. zygis*, *T. fragantissimus*, and *T. pulegioides* extracts was tested using a DPPH radical test, as described before [38]. Ascorbic acid was used as the positive control.

#### 3.6.2. Reducing Power Test

The ability of *T. zygis*, *T. fragantissimus*, and *T. pulegioides* (0.05–0.25 mg/mL) aqueous extracts to reduce iron (III) was assessed by the method previously described [39]. 2,6-Di-*tert*-butyl-4-methylphenol was used as the positive control.

### 3.7. Antimicrobial Activity

The antibacterial potential of the *Thymus* polar extracts was evaluated against five bacterial strains, including Gram-positive bacteria (*S. epidermidis* NCTC 11047 and *S. aureus* NCTC 6571) and Gram-negative bacteria (*S. typhimurium* NCTC 12023, *E. coli* NCTC 9001, and *P. aeruginosa* NCTC 10662) from the National Collection of Type Cultures, operated by Public Health England. All strains were cultured in Mueller–Hinton agar and incubated at 37 °C for 24 h.

The MIC and MBC of aqueous solutions of *T. zygis*, *T. fragantissimus*, and *T. pulegioides* were determined by the broth microdilution method, using an adapted method previously described by Afonso et al. [24]. Nisin, an antibacterial polypeptide approved as a food preservative, was used as the positive control [40].

### 3.8. Statistical Analysis

One-way analysis of variance (ANOVA) followed by Tukey’s test were used to detect any significant differences among different means. Alternatively, Student’s *t* test was used to determine the significant difference between two different samples. A *p*-value under 0.05 was assumed to indicate a significant difference. The results were analyzed using GraphPad Prism 6 (GraphPad Software, San Diego, CA, USA).

## 4. Conclusions

The nutritional composition, phenolic profile, antioxidant and antimicrobial activities of three species of *Thymus* grown under organic farming are reported within this study. Based on our results, it is possible to infer that these thyme species present a richness of polyunsaturated fatty acids and an interesting omega-6/omega-3 fatty acids ratio, along with high K/Na ratios that can contribute to cardiovascular protection. *T. pulegioides* presented the highest antioxidant capacity, consistent with its high content of polyphenols, which were mainly composed of rosmarinic acid and caffeoyl rosmarinic acid, also showing considerable levels of flavones (luteolin-*C*-glucoside, luteolin-*O*-glucuronide, and apigenin-*O*-glucuronide) and flavanones (eriodictyol-*O*-hexoside isomers). In addition, aqueous extracts obtained from these *Thymus* species, particularly *T. zygis*, showed antibacterial potential.

## Figures and Tables

**Table 1:** Nutritional value of the three *Thymus* plant species.

Total Content	*T. zygis*	*T. pulegioides*	*T. fragrantissimus*
Total Carbohydrates (g/100 g dw)	86.88 ± 0.51	81.22 ± 0.90	77.56 ± 0.16
Protein (g/100 g dw)	3.59 ± 0.13	8.28 ± 0.81	10.49 ± 0.13
Ash (g/100 g dw)	5.02 ± 0.25	8.24 ± 0.06	7.66 ± 0.22
Minerals (mg/100 g dw) *			
Na	7.6	7.4	9.0
K	1229.3	2185.6	1719.5
Ca	566.0	1039.4	865.6
Mg	119.8	148.3	241.8
Fe	4.4	1.9	7.8
Mn	9.8	15.7	6.9
Cu	0.1	0.1	0.3
Zn	2.1	1.7	2.7
Fat (g/100 g dw)	4.52 ± 0.12	2.27 ± 0.03	4.30 ±0.19
Fatty acids (relative %) *			
C12: 0	0.48	0.5	0.7
C14: 0	1.2	1.1	1.4
C16: 0	18.8	23.6	23.3
C18: 0	3.2	3.3	2.8
C18: 1n9	16.9	19.0	8.6
C18: 2n6c	32.6	20.4	19.3
C18: 3n3	15.6	27.6	36.2
C20: 0	2.8	1.8	2.6
C22: 1n9	8.3	2.7	5.0
SFA	26.5	30.2	30.9
MUFA	25.3	20.9	13.8
PUFA	48.3	48.9	55.3
PUFA/SFA	1.8	1.6	1.8
*n*-6/*n*-3	2.1	0.8	0.5
Caloric content (kcal/100 g dw)	402.50 ± 0.42	378.39 ± 0.08	390.86 ± 1.83

Lauric acid (C12: 0); Myristic acid (C14: 0); Palmitic acid (C16: 0); Stearic acid (C18: 0); Oleic acid (C18: 1n9c + t); Linoleic acid (C18: 2n6c); α-Linolenic acid (C18: 3n3); Arachidic acid (C20: 0); Erucic acid (C22: 1n9); * S.E.M. < 10%.

**Table 2:** Yield and total phenolic compounds of aqueous extracts of *T. zygis*, *T. pulegioides* and *T. fragrantissimus*.

	*T. zygis*	*T. pulegioides*	*T. fragrantissimus*
Yield of Extraction (%)	12.39 ± 0.60 ^a^	24.86 ± 1.71 ^b^	15.67 ± 4.56 ^a^
Total phenolic compounds (μg GAE/mg of extract)	287.86 ± 18.50 ^a^	390.94 ± 2.48 ^b^	287.08 ± 3.76 ^a^

Mean values ± S.D.; Statistical analysis was performed by one-way ANOVA, followed by Tukey’s test. In each line different letters (^a,b^) mean significant differences (*p* < 0.05).

**Table 3:** Identification and quantification of the compounds in the aqueous extracts of *T. zygis*, *T. pulegioides* and *T. fragrantissimus* by UHPLC-DAD-ESI-MS^n^.

NP	RT (min)	λmax	Compound	[M − H]^−^	MS^2^ Main Fragments ESI-MS^n^ (*m/z*)	*T. zig*	*T. pul*	*T. fragr*
1	1.3	298	Quinic Ac	191	111, 173, 93, 85	D	D	D
2	1.6	277	Syringic Ac hex	359	197, 179, 135	D	D	D
3	1.8	281	Danshensu	197	179, 73	D	D	D
4	1.9	283	Eriod di-*O*-hex	611	449, 287	-	D	-
5	2.4	271, 326	Api di-*C*-glc	593	473, 503, 575, 353	1.60 ± 0.32 ^a^	-	2.69 ± 0.50 ^a^
6	3.3	283	Eriod-*O*-hex (isom1)	449	287	-	9.43 ± 0.21	-
7		289, 321	CaffAc	179	135	8.46 ± 0.02 ^a^		2.54 ± 0.14 ^b^
8	3.4	285	SA F der	375	313, 179	D	-	D
9	3.7	283	Eriod-*O*-hex (isom2)	449	287	6.02 ± 0.94 ^a^	9.90 ± 0.19 ^b^	2.71 ± 0.03 ^c^
10	4.1	281, 342	Querc glcA	477	301	-	3.28 ± 0.03 ^a^	1.13 ± 0.25 ^b^
11	4.2	282, 342	Querc-*O*-hex	463	301	2.97 ± 0.90 ^a^	-	1.48 ± 0.02 ^b^
12	4.6	341	Lut-*C*-glc	447	357, 285, 327	4.86 ± 0.03 ^a^	8.27 ± 0.13 ^b^	2.00 ± 0.02 ^c^
13	5.6	281, 342	SA I/H	537	339, 493	-	D	-
14	6.2	258, 268, 342	Lut-*O*-di glc	593	285, 447	1.19 ± 0.01 ^a^	-	0.36 ± 0.02 ^b^
15	6.2	283	Nar-*O*-glc	433	271, 313	-	1.90 ± 0.97	-
16	6.8	282, 333	Scut-*O*-glcA	461	285, 175, 284, 257	D	D	-
17	7.0	254, 265, 345	Lut-*O*-glcA (isom1)	461	285	7.57 ± 0.05 ^a^	26.14 ± 0.78 ^b^	16.86 ± 0.21 ^c^
18	7.1	283	Eriod-*O*-glcA	463	287, 175, 151	-	D	D
19	7.3	282, 336	Querc-*O*-pent	433	301	D	-	-
20	7.6	288, 321	RA hex	521	359	D	-	D
21		254, 267, 344	Lut-*O*-glcA (isom2)	461	285	-	D	-
22	8.5	240, 339	Chrys-*O*-hex	461	299, 285	0.78 ± 0.01 ^a^	12.00 ± 0.15 ^b^	
23		281	CaffAc der	553	465, 311, 535, 357	-	-	D
24	8.7	283	Sagerinic Ac	719	359, 539, 521, 341	D	D	D
25	9.3	267, 334	Api-*O*-glcA	445	269, 175	D	9.20 ± 0.21 ^a^	1.76 ± 0.10 ^b^
26	9.6	290, 328	RA	359	223, 197, 179	62.36 ± 2.72 ^a^	81.65 ± 7.02 ^b^	81.04 ± 7.93 ^b^
27	9.8	287, 311	CaffRA (isom1)	537	493, 515, 357, 335, 519, 153	2.79 ± 0.24 ^a^	5.25 ± 0.19 ^b^	-
28	10.1	287, 328	SA B	717	519, 357, 555, 359	1.89 ± 0.20	-	-
29	10.7	290, 323	CaffRA (isom2)	537	493, 359, 519, 179	19.40 ± 0.76 ^a^	57.73 ± 1.95 ^b^	16.21 ± 0.54 ^c^
30	12.1	288, 322	CaffRA (isom3)	537	375, 493, 357, 359	D	D	D
31	12.5	287, 328	CaffRA (isom4)	537	439, 519, 357, 197, 493, 323, 331, 313	-	D	-
32	12.8	288, 323	CaffRA (isom5)	537	519, 359, 357, 339, 235, 493, 279, 207	-	D	-
					Total	119.90 ± 3.31 ^a^	225.79 ± 15.08 ^b^	126.65 ± 9.73 ^a^
					Caffeic acid and derivatives	94.89 ± 1.84 ^a^	144.63 ± 8.79 ^b^	101.12 ± 6.75 ^a^
					Flavones	16.01 ± 0.27 ^a^	55.62 ± 1.05 ^b^	21.67 ± 0.59 ^c^
					Flavonols	2.97 ± 0.90 ^a^	3.28 ± 0.03 ^a^	2.62 ± 0.25 ^a^
					Flavanones	6.02 ± 0.94 ^a^	19.93 ± 0.46 ^b^	2.71 ± 0.03 ^c^

NF-Number of peak represented in Figure 2; D-Detected; *T. zig*-*T. zygis*; *T. pul*-*T. pulegioides*; *T. fragr*-*T. fragrantissimus*; Ac-acid; Api-Apigenin; CaffAc-Caffeic acid; Caff-Caffeoyl; Chrys-Chrysoeriol; Der-Derivative; Eriod-Eriodictyol; Glc-Glucoside; GlcA-Glucuronide; Hex-Hexoside; Lut-Luteolin; Nar-Naringenin; Pent-Pentoside; Querc-Quercetin; RA-Rosmarinic acid; SA-Salvianolic acid; Scut-Scutellarein; Values are expressed as µg/mg extract; In each line different letters (^a,b,c^) mean significant differences (*p* < 0.05).

**Table 4:** Antioxidant and antibacterial properties of aqueous extracts from *T. zygis*, *T. pulegioides* and *T. fragrantissimus*.

	*T. zygis*	*T. pulegioides*	*T. fragrantissimus*	Standard
**Antioxidant properties**				
DPPH^● (1)^ (μg/mL)	12.65 ± 2.30 ^a,b^	9.50 ± 1.98 ^a,b^	12.87 ± 3.79 ^a^	6.90 ± 0.5 ^b^
Reducing Power ^(2)^ (μg/mL)	33.66 ± 1.93 ^a^	30.73 ± 1.48 ^a^	32.44 ± 4.27 ^a^	16.30 ± 1.5 ^b^
**Antibacterial properties** ^(3)^ (MIC/MBC, mg/mL)				
**G (+) bacteria**				
*Staphylococcus aureus*	1.13/1.13	5.75/5.75	3.75/3.75	0.25/0.25
*Staphylococcus epidermidis*	4.50/4.50	5.75/11.50	7.50/>7.50	<0.06/<0.06
**G (−) bacteria**				
*Salmonella typhimurium*	4.50/4.50	5.75/11.50	7.50/7.50	0.50/0.50
*Escherichia coli*	>4.5/>4.5	>11.50/>11.50	7.50/>7.50	0.50/0.50
*Pseudomonas aeruginosa*	4.5/>4.5	5.75/11.50	7.50/>7.50	0.50/1.0

^(1)^ Amount of extract required to reduce 50% of the 60 μM radical 2,2-diphenyl-1-picrylhydrazyl (DPPH^●^); Ascorbic acid was used as reference compound; ^(2)^ Amount of extract able to provide 0.5 of absorbance by reducing 3.5 μM Fe^3+^ to Fe^2+^; 2,6-Di-*tert*-butyl-4-methylphenol was used as reference compound; Mean values ± S.D.; Statistical analysis was performed by one-way ANOVA, followed by Tukey’s test. In each line different letters (^a,b^) mean significant differences (*p* < 0.05); ^(3)^ Nisin was used as reference compound. Mean values; MIC: minimum inhibitory concentration; MBC: minimum bactericidal concentration.

## Supplementary Materials

Supplementary materials can be found in a separate file: Figure S1: Pictures of *Thymus zygis* (a), *Thymus pulegioides* (b) and *Thymus fragrantissimus* (c); Figure S2: Extracted ion chromatograms and (inset) mass spectrum of ESI–MS^2^ of the corresponded ion of main phenolic compounds identified in *Thymus zygis*, *Thymus pulegioides* and *Thymus fragrantissimus* by UHPLC-DAD-ESI-MS^n^; Table S1: Linearity, LOD and LOQ of the standard compounds used as references.
