# Supplementary material for: Metabolites and Biological Activities of Thymus zygis, Thymus pulegioides, and Thymus fragrantissimus Grown under Organic Cultivation
Source: Molecules. 2018 Jun 22;23(7):1514. doi: 10.3390/molecules23071514 (PMC6099726; doi:10.3390/molecules23071514)
Supplement: Supplementary file 1 [file molecules-23-01514-s001.pdf]

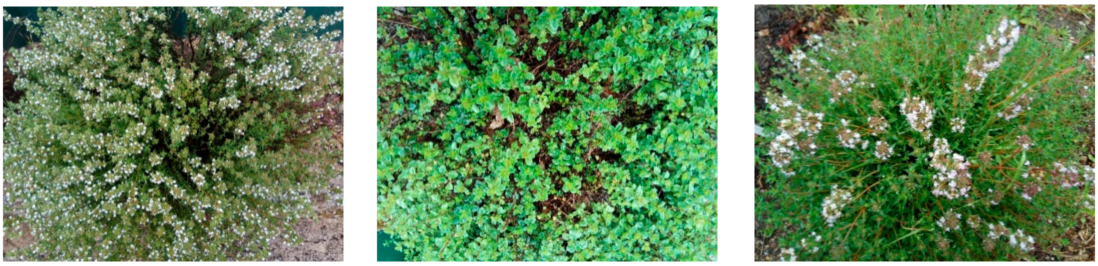

(a) (b) (c)

Figure S1. Pictures of *Thymus zygis* (a), *Thymus pulegioides* (b) and *Thymus fragrantissimus* (c). Numbers in the figure correspond to the UHPLC-DAD-ESI-MS^n peaks described in Table 3.

Table S1. Linearity, LOD and LOQ of the standard compounds used as references.

Standard Compound	Regression Equation	R^2	Range concentration (µg/mL)	LOD (µg/mL)	LOQ (µg/mL)
A-7O-G	y = 2E+07x - 4108	0.9994	1 - 50	2.1	6.3
E-7O-G	y = 1E+07x + 416	0.9997	1 - 100	3.1	9.2
L-7O-G	y = 2E+07x - 7697	0.9996	1 - 50	1.6	4.9
RA	y = 2E+07x - 67279	0.9970	26 - 517	52.1	158.0

LOD and LOQ were defined as 3.3 and 10 times the value of the regression error divided by the slope, respectively; L-7O-G: luteolin-7-*O*-glucoside; RA: rosmarinic acid; A-7O-G: apigenin-7-*O*-glucoside; E-7O-G: eriodictyol-7-*O*-glucoside; Injections were done in triplicate.

Rosmarinic acid (26)

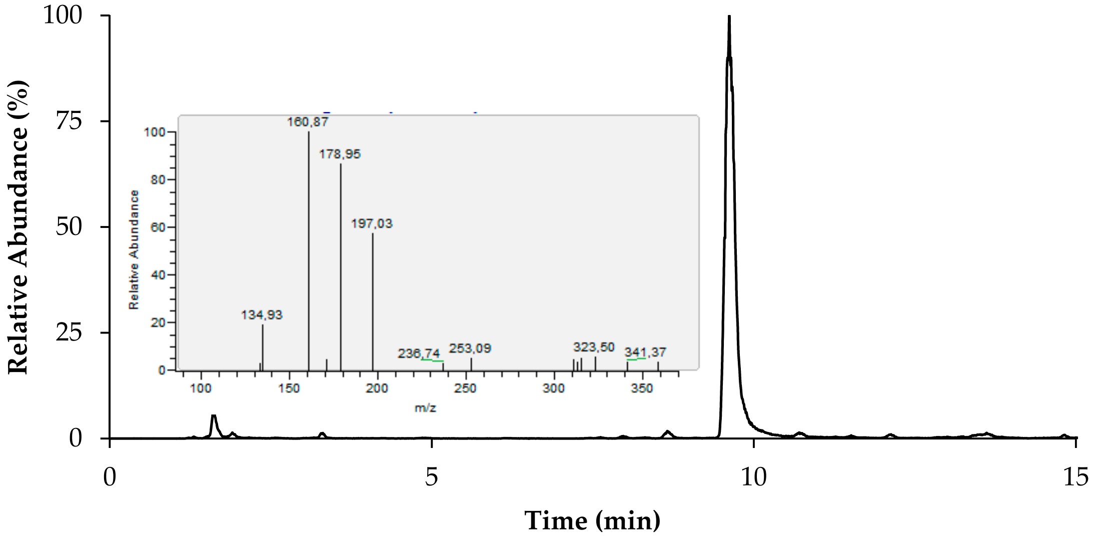

Caffeoyl rosmarinic acid (29)

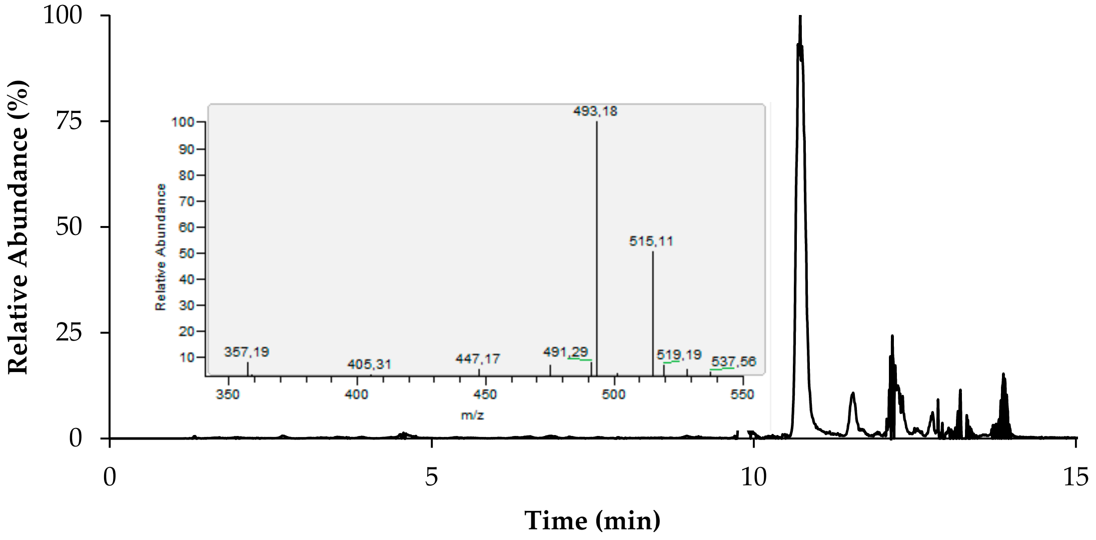

Luteolin-*C*-glucoside (12)

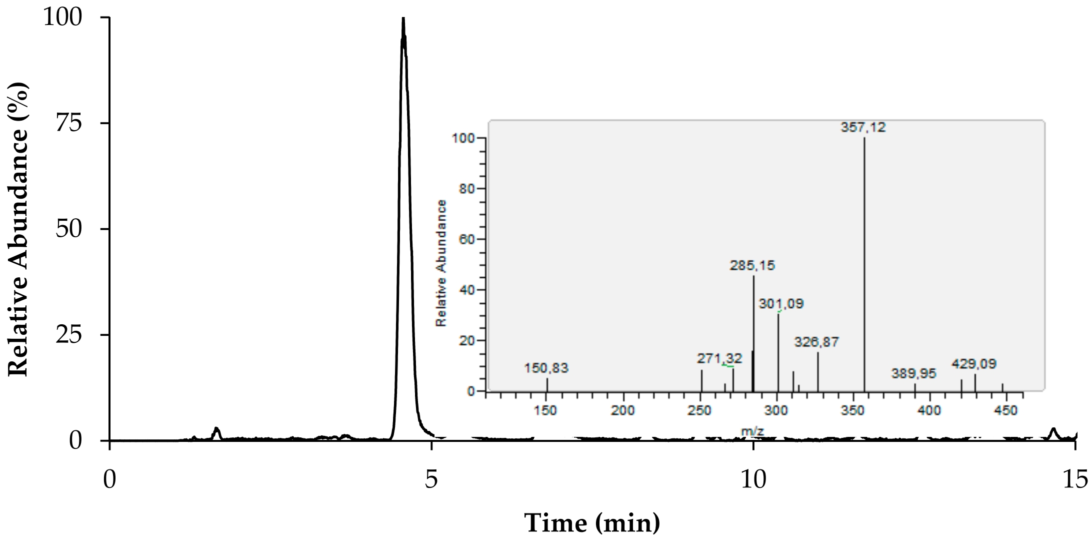

Luteolin-*O*-glucuronide (17)

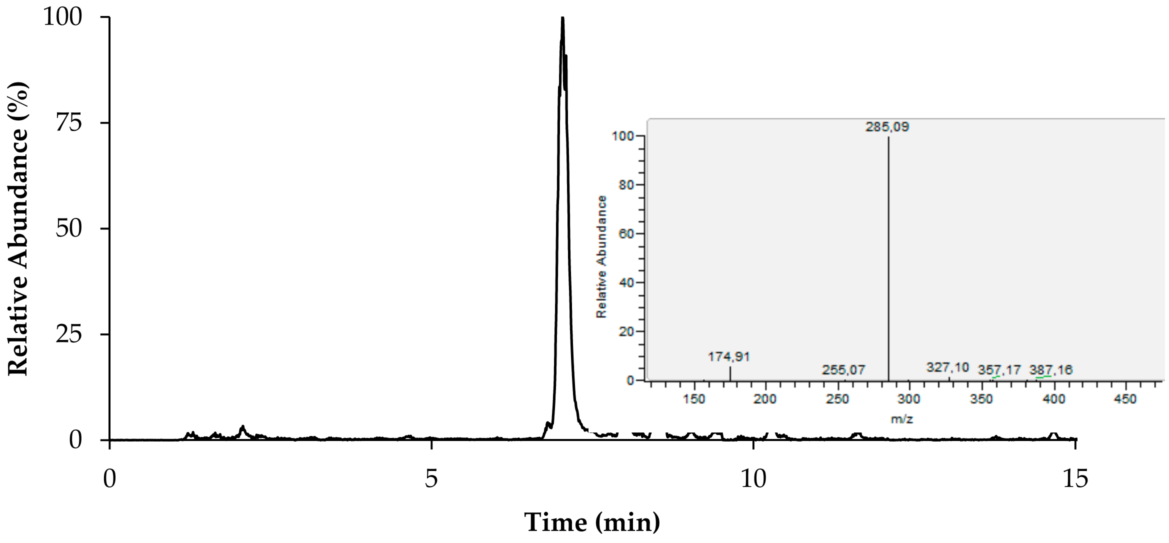

Apigenin-*O*-glucuronide (25)

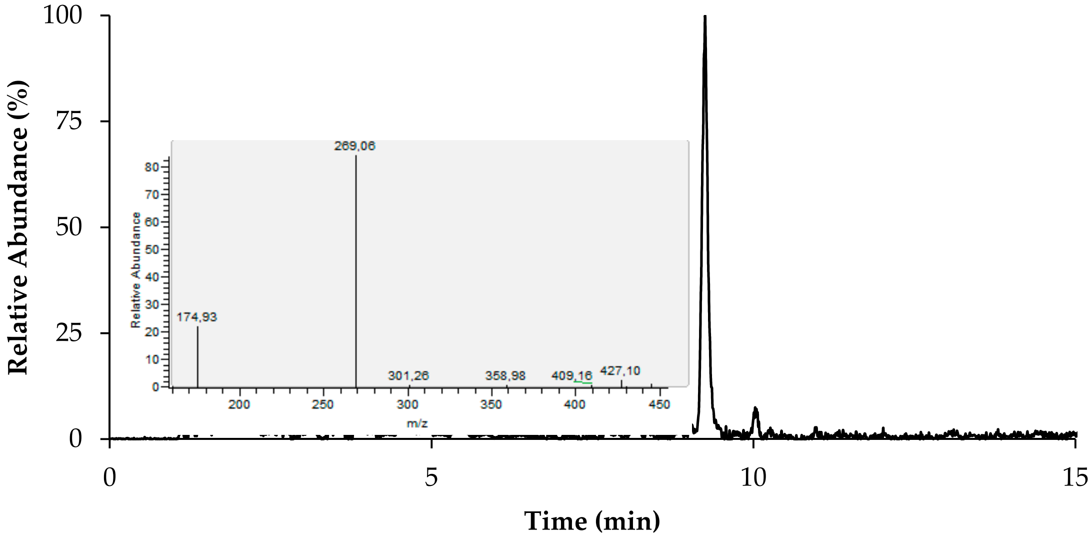

Eriodictyol-*O*-hexoside (9)

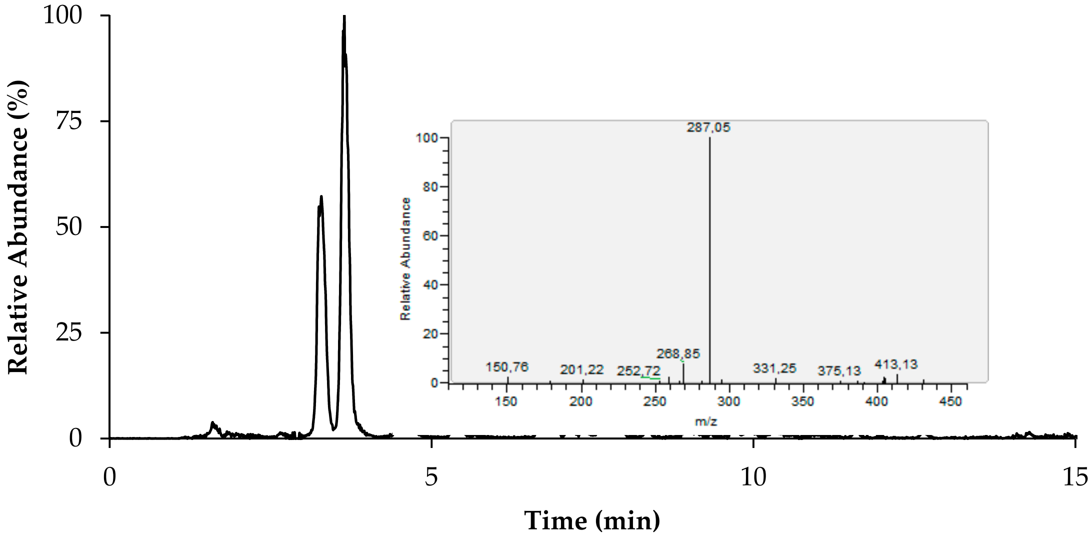

Figure S2. Extracted ion chromatograms and (inset) mass spectrum of ESI–MS^2 of the corresponded ion of main phenolic compounds identified in *Thymus zygis*, *Thymus pulegioides* and *Thymus fragrantissimus* by UHPLC-DAD-ESI-MS^n
